# Euthanasia

**Published:** 1906-05-05

**Authors:** 


### The Hospital

A Journal of

The Medical Sciences and Hospital Administration.

Vol. XL.—No. 1,023. SATURDAY, MAY 5, 1906.

### EUTHANASIA.

However conversant we may be with the independence of spirit by which the people of the United States of America are distinguished, it may fairly be assumed that the discussion now in progress amongst them, and which has found its way to Europe, concerning the advisability of terminating, by legitimate means, lives rendered intolerable by disease or pain, will excite surprise in the minds of a large proportion of our readers. The proposal is that a sufferer from incurable illness who is able to obtain certain certificates of the incurability of his condition, should be enabled to lay down his life with dignity, and under the shelter of legal forms and ceremonies provided for the occasion; making his testamentary dispositions in security, leaving no stain upon his memory to be transmitted to his descendants, and accepting, at the hands of the physician appointed for the purpose by the State, a painless passport to immortality. Nor does the suggestion stop here, for those who make it would also make provision for the extinction of the useless lives of some who were unable fully to weigh the question for themselves, of sufferers from chronic dementia, even of children hopelessly handicapped by disease. If the proposition were once seriously entertained, the area of lethal activity would, no doubt, be in some danger of gradual extension, even so far that a time might come at which useless persons, whether tramps or millionaires, might be in danger of being called upon to justify their own existences as a condition of being permitted to continue them.

If we approach the matter seriously, it is perhaps hardly possible to deny that the familiar phrase so often applied to death after long illness, the phrase "a happy release," has its roots very deeply laid in the human consciousness; or to deny that many medical practitioners, in cases of hopeless illness and of prolonged suffering, would not feel it to be a matter for serious regret if the dose of a sedative had proved to be too large for the mere purpose of alleviating pain. On the other hand, the general recognition that it is the supreme duty of the physician to save life, and the profound difficulty, if the legitimacy of abbreviating it were entertained as an open question, of deciding where to draw the line, have sufficed, in this country at least, to exclude the whole subject from discussion.

It may be said that it is never harmful to be compelled to take out established conclusions, or what pass for such, and to re-examine the grounds on which they rest; and, if the results of such examination were to confirm us in the common belief that men's lives are not at their own disposal, but are held by them in trust and cannot be blamelessly surrendered, then we should, of course, be compelled to regard the recent American proposals for abbreviating chronic and incurable illness as calling only for uncompromising rejection. In that case, should we not have more to do? Is it not the fact that our laws and customs place no impediments in the way of practices which are no less suicidal than the taking of a single overdose of morphia, although the effects are spread over a somewhat longer period? What is to be said about the man who drinks a bottle of brandy a day, but who does not roam the streets in a state of intoxication, or otherwise render himself a nuisance to the public? What is to be said about the man who was designed by Nature to live to be a hundred, but who habitually overeats himself, and dies at sixty because his excretory glands are replaced by connective tissue? The guilt attendant upon the premature extinction of life cannot be diminished by rendering the extinction gradual instead of sudden; and, among the criminal classes, slow poisoners have usually been regarded with peculiar abhorrence. The question is mainly one of evidence as to the character and the inevitable consequences of the course that is being pursued; and, in this respect, the evidence is being strengthened every day. We know far more about the consequences of certain excesses than was known to former generations, and are far better able to trace effects to their causes. We are in the presence of an amount of national physical deterioration which a high authority, after very careful investigation of the facts, has lately described as "a progressive evil, which threatens slow national extinction," and against which, as far as may be possible, we are surely bound to provide.

